# Bone metabolism characteristics and gender differences in patients with COPD: a cross-sectional study

**DOI:** 10.1186/s12890-022-02298-z

**Published:** 2023-01-03

**Authors:** Yuan Yuan, Wei Tian, Xiaohui Deng, Rui Yue, Xiaozhu Ge

**Affiliations:** Department of Geriatrics, Beijing Jishuitan Hospital, Beijing, 100035 People’s Republic of China

**Keywords:** Chronic obstructive pulmonary disease, Bone metabolism, Formation, Absorption, Gender

## Abstract

**Background:**

Gender differences in bone metabolism of people with chronic obstructive pulmonary disease (COPD) remain unclear. We aim to explore the characteristics of bone metabolism and its clinical significance for patients with COPD.

**Methods:**

A total of 564 cases (282 COPD cases and 282 controls) were preselected. Clinical and analytical characteristics of these cases were assessed. After excluding patients with other conditions known to disturb calcium metabolism, 333 patients (152 COPD cases and 181 controls) were identified. The medical records, indexes of bone turnover markers, serum calcium and phosphorus of the 333 patients were collected and their correlation was analyzed.

**Results:**

The 152 cases with COPD were 82.61 ± 7.745 years, 78.3% males, and the 181 age- and sex-matched control cases were 79.73 ± 11.742 years, 72.4% males. Levels of total procollagen type I amino-terminal propeptide (tPINP), osteocalcin (OC), serum calcium and phosphate were significantly lower (*P* < 0.001) while the level of parathormone (PTH) was significantly higher (*P* = 0.004) in COPD than in controls. The 25-hydroxycholecalciferol (25(OH)D_3_) was below the lower limit of normal value (LLN) in both groups, which was significantly lower in COPD males than in control males (*P* = 0.026). In COPD group, PTH level was significantly higher in females (*P* = 0.006), and serum P was lower in males (*P* = 0.006). The adjusted linear regression analysis showed that the levels of tPINP, OC and serum Ca were decreasing greatly in COPD group [β (95%CI) − 8.958 (− 15.255 to − 2.662), *P* = 0.005; − 4.584 (− 6.627 to − 2.542), *P* < 0.001; − 0.065 (− 0.100 to − 0.031), *P* < 0.001]. Besides, smoke exposure, gender (male) were also related to hypocalcemia [β (95%CI) − 0.025 (− 0.045 to − 0.005), *P* = 0.017; − 0.041 (− 0.083 to − 0.001), *P* = 0.047], and 25(OH)D_3_ was correlated with serum calcium, phosphorus, and PTH [β (95%CI) 15.392(7.032–23.753), *P* < 0.001; − 7.287 (− 13.450 to − 1.124), *P* = 0.021; − 0.103(− 0.145 to − 0.061), *P* < 0.001], and female was more likely to have secondary hyperparathyroidism [β (95%CI) 12.141 (4.047–20.235), *P* = 0.002].

**Conclusion:**

COPD patients have remarkably low bone turnover (indicated by OC) and impaired bone formation (low tPINP), and they are also more prone to low calcium. Smoking and male may play roles in the formation of hypocalcemia, and the secondary hyperparathyroidism is more significant in COPD women. There may be gender differences in bone metabolism abnormalities and their mechanisms of COPD. The conclusion above still need further research and demonstration.

## Introduction

Research showed that osteoporosis occurs in about 24–44% of COPD patients [1, 2]. Due to the lack of specific clinical symptoms in the early stage of osteoporosis, fragile fracture has become the most common basis for first diagnosis [3], and can not only significantly increase the short-term (within 3 months after fracture) all-cause mortality of patients, but also lead to markedly higher long-term disability incidence, imposing a heavy burden on families and society [4].

Osteoporosis occurs mainly in females, and the incidence in postmenopausal females is about 5–10 times higher than that in males [5, 6]. In contrast, COPD patients are mainly males [7] suggesting that metabolic activity is markedly different between males and females with COPD. However, few clinical studies on gender differences in bone metabolism have been reported to date.

Bone turnover markers (BTM_S_) have been widely used in clinical practice in recent years. As good indicators of real-time bone metabolism level, they provide a significant reference for determining the anti-osteoporotic treatment protocols and evaluation of treatment effect. Some studies have shown that BTM_S_ also have good potential for early osteoporosis screening [7, 8].

In view of the lack of data on biomarkers in male and female patients with COPD, we collected demographic information and BTM data of COPD and non-COPD patients, and then conducted a comparative analysis between genders within and between groups, in an attempt to increase the understanding of the factors influencing bone metabolism in COPD patients.

## Methods

This was a cross-sectional, retrospective study of bone metabolism characteristics and gender differences in patients with COPD and non-COPD controls. All patients’ data were used anonymously. Informed consents were waived for this study due to its retrospective and observational nature.

We collected medical records of two groups of patients, who were outpatients or admitted to the health care, geriatrics or general medical department in Beijing Jishuitan Hospital from January, 2016 to December, 2020, and aged ≥ 60yrs old. *The COPD Group* included people: 1) diagnosed by the criteria of FEV1/FVC < 70% by spirometry [7], or 2) with physician diagnosis of COPD, according to the typical clinical symptoms of chronic bronchitis (cough and sputum production on most days for at least 3 months in the year for at least 2 consecutive years), signs of emphysema, in need of regularly used medication for treatment [9, 10]; *The Control Group* included people: diagnosed without COPD, with sex and age being matched on a 1:1 basis.

Exclusion criteria were the following: 1) with acute diseases, such as acute infection or trauma, e.g. pneumonia, active pulmonary tuberculosis, and bone fracture; 2) whose chronic diseases were in unstable stage, e.g. COPD exacerbation; 3) suffering from endocrine and metabolic, blood system, chronic liver, chronic kidney, or connective tissue diseases; 4) taking gastrointestinal resection or long-term oral glucocorticoids, immunosuppressants, sex hormones, thyroid hormones or other drugs affecting bone metabolism; 5) taking inhalation /oral / intravenous corticosteroids for COPD maintenance / exacerbation treatment in the previous year; and 6) BTMs values or medical records were incomplete.

Initially, a total of 564 study subjects (282 COPD cases and 282 controls) were considered as eligible ones. After excluding unqualified cases, 333 participants (152 COPD cases and 181 controls) were finally enrolled in our study. Of the 152 COPD cases, 122 patients were diagnosed with the pulmonary function findings, FEV_1_/FVC was 0.55 ± 0.09, and the GOLD I (FEV1/predicted ≥ 80%) 26.2% (32/122), GOLD II (FEV_1_/predicted 50–79%) 54.1% (66/122), GOLD III (FEV_1_/predicted 30–49%) 16.4% (20/122), GOLD IV (FEV_1_/predicted < 30%) 3.3% (4/122). Altogether 30 COPD cases were fulfilled with the second diagnostic method above by clinical comprehensive judgment. Of the 152 cases, the average time of exacerbation last year was 0 (IQR 0–0), in which 11 patients had the exacerbation history of COPD, but weren’t hospitalized.

### Bone turnover markers (BTMs)

Biochemical markers of bone formation and resorption and their normal ranges are as follows: 1) total procollagen type I amino-terminal propeptide (tPINP) (15.3–52.7 ng/mL), **s**ynthesized by osteoblasts and reflecting the speed of type I collagen synthesis and bone transformation, and the most sensitive marker of bone formation; 2) osteocalcin (OC) (12–42 ng/mL in males, 15–46 ng/mL in postmenopausal females), the most abundant and important non-collagenous protein in bone matrix. When bone remodeling is balanced (bone formation is coupled with bone resorption), OC is an indicator of bone turnover; when the bone resorption rate exceeds the formation rate (uncoupled state), resulting in bone volume reduction, OC becomes an indicator of bone formation; 3) β-isomerized C-terminal telopeptides (β-CTX) (< 0.854 ng/mL in males, < 1.008 in postmenopausal females) is a degradation product of type I collagen in the process of bone metabolism, and serves as a good marker of bone resorption.

Serum calcium and phosphorus, calcium- and phosphate- regulating hormones, and their normal ranges are as follows: 1) 25-hydroxycholecalciferol (25(OH)D_3_) (20–40 ng/mL); 2) parathormone (PTH) (15–65 pg/mL); 3) Calcium (Ca) 2.20–2.55 mmol/L; 4) Phosphate (P) 0.81–1.65 mmol/L.

BTMs of all patients were drawn on an empty stomach between 06:00 to 09:00 in the morning, and were measured using a Roche COMBAS e601 autoanalyzer (Roche Diagnostics, Basel, Switzerland) with the standard Cobas kit (Roche, Shanghai, China).

### Statistical analysis

Data were analyzed using SPSS 28.0 (IBM Corp., Armonk, NY, USA). All the measurement data were tested for normality with the Kolmogorov–Smirnov test. Descriptive data were expressed by mean ± standard deviation (X ± S) when in a normal distribution or median (inter-quartile range; IQR) when in a skewed distribution, and frequency data were expressed by rate. For normally distributed samples, ANOVA was used for comparing BTMs differences between individuals with and without COPD, and between-genders. Non-normally distributed data were analyzed using a nonparametric test (Mann–Whitney U test). Frequency data were compared using the chi-square test. In the analysis of the correlation between BTMs and COPD, we adopted the linear regression analysis. TPINP, OC, β-CTX, 25(OH)D_3_, PTH, serum Ca and P were taken as dependent variables respectively, first, the COPD unadjusted regression analysis was conducted, then the adjusted regression analysis was conducted, in which the independent variables included gender, age, chronic diseases, with or without COPD and smoking history (pack-year tobacco exposure). Considering the multiple confounding effects of vitamin D, serum Ca and P on other BTMs, we also included these three items as independent variables in the regression analysis of other four BTMs. The significance level was set at a *P* value < 0.05.

## Results

Demographic and clinical characteristics were shown in Table 1. The main characteristics were: 152 COPD cases 82.61 ± 7.745 years, 78.3% males (119 / 152); 181 control cases 79.73 ± 11.742 years, 72.4% males (131 / 181). There were no significant differences between groups in age or sex ratio. Besides, no significant differences were found in prevalence rates of comorbidities between two groups, except chronic pulmonary diseases without COPD (*P* = 0.004), and pack-year tobacco exposure (*P* < 0.001).

**Table 1:** Descriptive characteristics of the participants

Variables	ALL	COPD	Non-COPD	*Z/X*^*2*^	*P*
	n = 333	n = 152	n = 181		
Age (years)	81.01 ± 9.515	82.61 ± 7.745	79.73 ± 11.742	− 1.810	0.081
Gender(Male)	250 (75.08)	119 (78.29)	131 (72.38)	1.544	0.214
Smoking					
Pack-years (PY) = 0	179 (53.75)	56 (36.73)	123 (67.77)	25.631	< 0.001*
0–29.9PY	72 (21.62)	44 (29.25)	28 (15.70)		
⩾30PY	82 (24.62)	52 (34.01)	30 (16.53)		
Components of chronic diseases^#^					
Atherosclerosis	70 (21.02)	26 (17.11)	44 (24.31)	0.071	0.194
Coronary heart disease	22 (6.61)	8 (5.26)	14 (7.73)	0.065	0.366
Hypertension	22 (6.61)	10 (6.58)	12 (6.63)	0.001	0.985
Arrhythmia	7 (2.10)	4 (2.63)	3 (1.66)	0.034	0.537
Pulmonary diseases without COPD	6 (1.80)	6 (3.95)	0 (0)	8.515	0.004*
Old cerebral infarction	6 (1.80)	4 (2.63)	2 (1.10)	1.088	0.297

Continuous variables are expressed as means ± standard deviation (SD), categorical data presented as whole numbers (percentage), and a *p*-value < 0.05 is considered statistically significant and indicated by an asterisk (*)

^#^ Atherosclerosis included 32 cases of carotid atherosclerosis and 38 cases of cerebral atherosclerosis; Arrhythmias included 3 cases of sick sinus syndrome, 2 cases of frequent atrial premature beats, 1 case of atrial fibrillation, and 1 case of sinus bradycardia; Other pulmonary diseases included 4 cases of bronchiectasis, 1 case of pulmonary interstitial fibrosis, and 1 case of bronchial asthma. *COPD* Chronic obstructive pulmonary disease

### Comparison of BTMs between groups

The average levels of BTMs were shown in Table 2. OC was below the lower limit of normal value (LLN) in the COPD group, and 25(OH)D_3_ was below LLN in both groups. Compared with the control group, the levels of tPINP, OC and serum Ca, P in the COPD group were significantly lower (all *P* < 0.001) and PTH significantly higher (*P* = 0.004). There was a trend of higher β-CTX and lower 25(OH)D_3_ in COPD than the control without statistical difference (*P* = 0.499, *P* = 0.092).

**Table 2:** Comparison of bone turnover markers (BTMs) between groups and genders

BTMs	Gender	ALL	COPD	Non-COPD	*Z*	*P*
		n = 333	n = 152	n = 181		
tPINP (ng/ml)	Total	41.56 ± 21.85	37.29 ± 23.02	45.16 ± 20.18	− 4.516	< 0.001^*^
	Male	39.00 ± 22.54	37.07 ± 24.04	42.07 ± 19.69	− 2.818	0.005^*^
	Female	45.15 ± 20.39	38.08 ± 19.18	47.35 ± 20.33	− 2.237	0.025^*^
β-CTX (ng/ml)	Total	0.48 ± 0.53	0.51 ± 0.74	0.45 ± 23.29	− 0.675	0.499
	Male	0.48 ± 0.66	0.52 ± 0.82	0.43 ± 22.46	− 0.231	0.817
	Female	0.47 ± 0.26	0.49 ± 0.33	0.46 ± 0.24	− 0.203	0.839
OC (ng/ml)	Total	14.89 ± 7.37	12.20 ± 6.88	17.15 ± 7.01	− 6.933	< 0.001^*^
	Male	13.05 ± 6.43	11.85 ± 6.71	14.96 ± 5.47	− 4.174	< 0.001^*^
	Female	17.47 ± 7.84	13.48 ± 7.44	18.71 ± 7.57	− 3.663	< 0.001^*^
25(OH)D_3_ (ng/ml)	Total	17.57 ± 8.96	16.75 ± 8.95	18.25 ± 8.94	− 1.685	0.092
	Male	18.15 ± 9.14	17.02 ± 8.97	19.89 ± 9.20	− 2.233	0.026^*^
	Female	16.78 ± 8.68	15.7 ± 8.96	17.09 ± 8.60	− 0.997	0.319
PTH (pg/ml)	Total	49.26 ± 26.24	54.51 ± 30.94	44.85 ± 20.62	− 2.886	0.004^*^
	Male	49.47 ± 26.24	52.04 ± 29.77	45.4 ± 18.83	− 1.000	0.317
	Female	48.97 ± 26.36	63.4 ± 33.80	44.47 ± 21.88	− 3.505	< 0.001^*^
Ca (mmol/L)	Total	2.29 ± 0.15	2.23 ± 0.15	2.34 ± 0.11	3.632	< 0.001^*^
	Male	2.28 ± 0.15	2.21 ± 0.16	2.33 ± 0.12	1.539	< 0.001^*^
	Female	2.33 ± 0.12	2.26 ± 1.45	2.38 ± 0.08	4.456	< 0.001^*^
P (mmol/L)	Total	1.04 ± 0.19	0.97 ± 0.19	1.11 ± 0.18	1.646	< 0.001^*^
	Male	1.02 ± 0.21	0.95 ± 0.19	1.10 ± 0.19	1.202	< 0.001^*^
	Female	1.10 ± 0.15	1.05 ± 0.15	1.15 ± 0.14	0.018	0.003^*^

Continuous variables are expressed as means ± standard deviation (SD), and a *p*-value < 0.05 is considered statistically significant and indicated by an asterisk (*)

*COPD* Chronic obstructive pulmonary disease, *tPINP* total procollagen type I amino-terminal propeptide, *β-CTX* β-isomerized C-terminal telopeptides, *OC* Osteocalcin, *25(OH)D*_*3*_ 25-hydroxycholecalciferol, *PTH* Parathormone, *Ca* calcium, *P *phosphate

### Analysis of gender differences between and within groups

As shown in Table 2, levels of tPINP and OC were significantly lower in COPD males and COPD females (all *P* < 0.005). The level of 25(OH)D_3_ in males with COPD was significantly lower than males in controls (*P* = 0.026), while the PTH level of females with COPD was significantly higher than the other females (*P* < 0.001).

Further comparison within the COPD group showed that (Table 3), the PTH level was significantly higher in females (*P* = 0.006), and serum P was lower in males (*P* = 0.006). In the control group, the level of OC in females was higher (*P* = 0.015), and serum Ca was lower in males (*P* = 0.029).

**Table 3:** Comparison of bone turnover markers (BTMs) between different genders within two groups

BTMs (*Z/P*)	tPINP (ng/ml)	β-CTX (ng/ml)	OC (ng/ml)	25(OH)D_3_ (ng/ml)	PTH (pg/ml)	Ca (mmol/L)	P (mmol/L)
COPD (n = 152)	1.169/0.739	0.739/0.645	0.795/0.552	0.894/0.402	1.706/0.006^*^	− 1.545/0.060	− 2.770/0.006^*^
Non-COPD (n = 181)	− 0.937/0.349	− 0.020/0.984	− 2.422/0.015^*^	− 1.642/0.101	− 1.892/0.058	− 2.201/0.029^*^	− 1.771/0.079

Mann-Whitney U test was used, and a *p*-value < 0.05 is considered statistically significant and indicated by an asterisk (*)

*COPD* Chronic obstructive pulmonary disease, *tPINP* total procollagen type I amino-terminal propeptide, *β-CTX* β-isomerized C-terminal telopeptides, *OC* osteocalcin, *25(OH)D*_*3*_ 25-hydroxycholecalciferol, *PTH* Parathormone, *Ca* calcium, *P* phosphate

### Linear regression analysis of BTMs and COPD

Firstly, we conducted an unadjusted linear regression analysis. Then the adjusted analysis was carried out. Results showed that (Table 4), tPINP, OC and serum Ca were decresing greatly in the COPD group[β (95%CI) − 8.958 (− 15.255 to − 2.662), *P* = 0.005; − 4.584 (− 6.627 to − 2.542), *P* < 0.001; − 0.065 (− 0.100 to − 0.031), *P* < 0.001]. Besides, smoke exposure, gender (male) were also related to hypocalcemia [β (95%CI) − 0.025 (− 0.045 to − 0.005), *P* = 0.017; − 0.041 (− 0.083 to − 0.001), *P* = 0.047]. 25(OH)D_3_ was significantly correlated with serum calcium, phosphorus and PTH [β (95%CI) 15.392(7.032–23.753), *P* < 0.001; − 7.287 (− 13.450 to − 1.124), *P* = 0.021; − 0.103(− 0.145 to − 0.061), *P* < 0.001], and females had higher PTH level [β (95%CI) 12.141 (4.047–20.235), *P* = 0.002].

**Table 4:** Linear regression analysis of the correlation between BTMs and COPD

Dependent variables	UNADJUSTED			Independent variables	ADJUSTED		
	β (95%CI)	t	*P*		β (95%CI)	t	*p*
tPINP	− 7.870 (− 12.527 to − 3.212)	− 3.324	< 0.001*	COPD	− 8.958 (− 15.255 to − 2.662)	− 2.802	0.005﻿*
β-CTX	0.066 (− 0.050 to 0.181)	1.122	0.263	COPD	− 0.025 (− 0.145 to 0.039)	− 1.018	0.310
OC	− 4.952 (− 6.458 to − 3.448)	− 6.474	< 0.001﻿*	COPD	− 4.584 (− 6.627 to − 2.542)	− 4.421	< 0.001﻿*
25(OH)D_3_	− 1.505 (− 3.451 to 0.441)	− 1.521	0.129	COPD	− 0.293 (− 2.813 to 2.228)	− 0.229	0.819
				Ca	15.392(7.032–23.753)	3.627	< 0.001﻿*
				P	− 7.287 (− 13.450 to − 1.124)	− 2.329	0.021﻿*
				PTH	− 0.103(− 0.145 to − 0.061)	− 4.831	< 0.001﻿*
PTH	9.654 (4.061–15.246)	3.396	< 0.001﻿*	COPD	0.828 (− 6.379 to 8.035)	0.226	0.821
				Gender (F)	12.141 (4.047–20.235)	3.142	0.002﻿*
				Serum P	− 21.362 (− 38.570 to − 4.154)	− 2.249	0.015﻿*
Ca	− 0.114 (− 0.144 to − 0.084)	− 7.455	< 0.001﻿*	COPD	− 0.065 (− 0.100 to − 0.031)	− 3.741	< 0.001﻿*
				Smoking	− 0.025 (− 0.045 to − 0.005)	− 2.412	0.017﻿*
				Gender (M)	− 0.041 (− 0.083 to − 0.001)	− 2.026	0.047﻿*
				Serum P	0.262 (0.177–0.347)	6.088	< 0.001﻿*
P	− 0.144 (− 0.185 to − 0.102)	− 6.853	< 0.001﻿*	COPD	0.021 (− 0.040 to 0.082)	0.682	0.496
				Age	− 0.003 (− 0.006 to − 0.001)	− 2.587	0.010﻿*

A *p*-value < 0.05 is considered statistically significant and indicated by an asterisk (*). ﻿**1**) Serum calcium, serum phosphorus and five bone metabolism markers were included in the linear regression analysis as dependent variables respectively. The independent variable in the unadjusted analysis was only COPD. In adjusted analysis, the independent variables included continuous variables (age, serum calcium, serum phosphorus, 25(OH)D_3_), 2 categorical variables (gender, whether atherosclerosis, whether coronary heart disease, whether hypertension, whether arrhythmia, whether pulmonary diseases except COPD, whether COPD, whether old cerebral infarction), 3 categorical variables (package-year smoke exposure, “0PY” = 0, “0–29.9PY” = 1, “⩾30PY” = 2); **2**) Serum calcium, phosphorus and 25(OH)D3 did not included in their own analysis as independent variables, and dependent and independent variables with linear correlation will not be listed repeatedly; **3**) Confounding variables with *P* < 0.05 values were also listed in the table. *COPD *Chronic obstructive pulmonary disease, *tPINP *total procollagen type I amino-terminal propeptide, *β-CTX *β-isomerized C-terminal telopeptides, *OC* osteocalcin, *25(OH)D3* 25-hydroxycholecalciferol, *PTH *Parathormone﻿, *Ca *calcium, *P *phosphate, *M *male, *F *female

## Discussion

Many studies have shown a comorbidity between COPD and osteoporosis [2, 11], yet few have reported on the similarities and differences between BTMs in COPD patients and controls. BTMs indicate that osteoporosis can be categorized as high conversion (postmenopausal osteoporosis, type I) and low conversion (senile osteoporosis, type II) [12]. Bone loss rate is faster in the former than the latter and osteoporosis with low conversion rate has a higher risk of fracture due to slow bone formation and decreased bone mass.

This study found significantly lower mean levels of tPINP and OC in patients with COPD, and strong correlation between tPINP, OC and COPD. The increase of β-CTX in COPD group did not show statistical difference. We may infer from this finding that COPD-induced abnormality in bone metabolism may be manifested mainly in the reduction of bone turnover rate and osteogenic dysfunction. For females with COPD, its inhibitory effect on bone formation rate may offset or even exceed the high bone turnover rate caused by menopause. The mechanism by which COPD affects bone metabolism remains unclear. Possible causes may be that the complications of low activity in patients with COPD weakened the positive stimulation effect of muscle-bone unit [13, 14] and hypoxia in COPD which may decrease ferritinophagy and autophagy flux, inhibited RANKL-induced ferroptosis in osteoclasts, and eventually accelerated bone loss [15].

Vitamin D deficiency was also very common in these cases, which could not only be found in the COPD group, but also in the controls. Furthermore, vitamin D deficiency seemed more serious in COPD, especially in COPD females. Why did the COPD patients lack Vitamin D badly? Till now, there was no definite conclusion about the cause and effect of COPD and vitamin D deficiency, or the possible interaction between them. However, it is certain that COPD can induce systemic inflammation, while vitamin D deficiency may maintain and aggravate this situation, forming a vicious circle [16, 17]. In other words, if vitamin D is supplemented, the rate of moderate/ severe COPD exacerbation may be reduced to a certain extent [18].

We also found decrease of calcium in COPD group, and smoking and male promote hypocalcemia probably. No research on the correlation between cigarette smoking and hypocalcemia was found, previous studies seem to focus mainly on the correlation between smoking and PTH, and the conclusions were inconsistent. One animal study [19] showed that cigarette smoke inhalation increased serum levels of the hormone calcitoninthe, decreased in lung tissue immunoreactive calcitonin content and caused hypocalcemia. However, another study [20] showed that smokers had lower serum PTH levels than non-smokers, and there was no association between number of cigarettes smoked and serum PTH. Further consideration, do smoking and sex hormones have a synergistic effect on serum calcium levels or even bone metabolism? Some researches [21, 22] on the correlation between smoking and hormones showed a positive correlation between cigarette smoking and serum androgen concentration, but there was no association between either serum oestrogens and cigarette smoking. Therefore, whether hypocalcemia in COPD patients relating to smoking and abnormal androgen levels simultaneously needs further study. But it seems to be verified that, hypocalcemia may be related to the disease progression, respiratory infection rate, and hospital stay of patients with AECOPD [23].

As is known that, excessive secretion of PTH can increase the bone conversion rate and enhance the decomposition and absorption of bone, resulting in osteoporosis [12]. The present study showed that mean PTH level is higher in COPD, especially higher in COPD females. Combined with the results of linear regression analysis, it is considered that secondary hyperparathyroidism is related to hypocalcemia and relative vitamin D dificiency in patients with COPD, which may leads to hypophosphatemia. The above calcium and phosphorus metabolism disorder aggravates the decrease of bone mineral density in patients with COPD.

## Limitations

The present study has some limitations. First, it is a single center cross-sectional study, and the included population is limited in representation, which may affect the reliability of results, so some conclusions still need to be further verified. Second, although we find that decreasing OC and tPINP levels are significantly correlated with COPD, smoking and male are strongly correlated with hypocalcemia, and secondary hyperparathyroidism seems the most serious in female COPD patients. Factors such as menopause, hormone levels, corticosteroids use one year ago or even earlier, vitamin D susceptibility and lifestyle may complicate the mechanisms of osteopenia and osteoporosis in males and females with COPD. Therefore, the role of the BTMs in bone metabolism monitoring, assessment of treatment efficacy and disease prognosis for COPD patients with different genders still needs to be further studied.

## Conclusion

Patients with COPD have lower bone turnover and bone formation than those without COPD, and the tPINP and OC can be used as good indicators of bone metabolism in COPD persons. Smoking and male may be causes of hypocalcemia in COPD, whose specific mechanism remains to be studied. Female patients with COPD are more likely to have secondary hyperparathyroidism, which needs more clinical attention.

## Data Availability

With the permission of the corresponding authors, we can provide participant data without names and identifiers. The corresponding authors have the right to decide whether to share the data based on the research objectives and plan provided. Please contact correspondence author for data requests.

## Abbreviations

COPD: Chronic obstructive pulmonary disease
BTMs: Bone turnover markers
tPINP: Total procollagen type I amino-terminal propeptide
β-CTX: β-Isomerized C-terminal telopeptides
OC: Osteocalcin
25(OH)D_3_: 25-Hydroxycholecalciferol
PTH: Parathormone
Ca: Calcium
P: Phosphate
LLN: Lower limit of normal value
FEV_1_: Forced expiratory volume in 1 s
FVC: Forced vital capacity
